# A Comparative Analysis of Magnesium Sulfate Administered Intravenously Versus Perineurally as an Additive to Ropivacaine in Supraclavicular Brachial Plexus Block Under Ultrasound Guidance: A Randomized Clinical Trial

**DOI:** 10.7759/cureus.72944

**Published:** 2024-11-03

**Authors:** Sudheer Ramegowda, Karthik GS, Mahesh Chandra, Lini Rajan, Dilip Kumar M, Prem Kumar R

**Affiliations:** 1 Anaesthesiology, Rajarajeswari Medical College and Hospital, Bangalore, IND; 2 Anaesthesiology and Critical Care, Rajarajeswari Medical College and Hospital, Bangalore, IND

**Keywords:** forearm surgery, intravenous, magnesium sulphate, peineural block, perineurial magnesium sulphate, randomised controlled trial, ropivacaine, supraclavicular brachial plexus block, ultrasound guided nerve block, upper limb surgery

## Abstract

Background and objective

Ultrasound-guided supraclavicular brachial plexus block has revolutionized the anesthesia practice, and a single injection can facilitate the rapid onset of anesthesia. Ropivacaine has replaced bupivacaine due to its enhanced cardiovascular and neurological safety profile. Several clinical investigations have demonstrated that magnesium sulfate administration during peripheral nerve blocks can reduce the anesthetic requirements and postoperative analgesic consumption. In this study, we aimed to compare the analgesic efficacy of perineural magnesium sulfate versus intravenous (IV) magnesium sulfate as an adjuvant to ropivacaine in patients undergoing upper limb orthopedic surgeries under supraclavicular brachial plexus block. The secondary objectives included analyzing the duration and onset of sensorimotor blockade, total doses of rescue analgesic administered, hemodynamic profile, and adverse effects.

Methodology

We conducted a prospective randomized study involving 50 patients with the American Society of Anaesthesiologists (ASA) grade I and II who were aged 18-60 years and scheduled for elective upper limb orthopedic surgeries to treat both-bone forearm fractures. We adopted a single-blinded study design and patients were randomly allocated into two groups based on a sealed opaque envelope technique. Both groups received 0.75% ropivacaine 20 ml; in addition, Group IV Mg received 2 mL of normal saline and Group perineural Mg received 150 mg of magnesium sulfate (2 ml) as additives, amounting to a total of 22 ml for supraclavicular brachial plexus block. Group IV Mg received an injection of magnesium sulfate 150 mg in 100 ml of isotonic saline IV whereas Group perineural Mg received 100 ml of isotonic saline IV 30 minutes before the administration of supraclavicular brachial plexus block.

Results

Both groups were statistically comparable in terms of all demographic variables, ASA grading, and duration of surgery. Duration of analgesia was prolonged in Group perineural Mg (616.48 ± 92.396 min) vs. Group IV Mg (459.81 ± 82.984 min) (p = 0.001). The duration of sensory and motor blockade was significantly higher in Group perineural Mg when compared to Group IV Mg (p<0.001). Intraoperative hemodynamic parameters were comparable between the groups, and no side effects were reported in either of the groups.

Conclusions

Based on our findings, magnesium sulfate administered perineurally as an additive in the supraclavicular brachial plexus block is associated with a superior duration of analgesic effect when compared to the IV route. Perineural magnesium sulfate is also more effective in increasing the duration of sensorimotor blockade.

## Introduction

Supraclavicular brachial plexus block is a commonly used technique under ultrasound guidance for upper limb orthopedic surgeries to anesthetize mid-humeral level down to the hand. The anatomical advantage of this block has been well elucidated, as the brachial plexus elements are compactly grouped in this area. This promotes a very rapid onset of anesthesia and prolonged analgesia in the postoperative period without any adverse effects. This technique also reduces and eliminates the need for any additional intraoperative and postoperative opioid analgesics, facilitating enhanced recovery, minimizing hospital stay, and promoting better patient comfort and surgeon satisfaction. The economic burden to the patient is also considerably lower, which favors its use in daycare surgeries when compared to general anesthesia [1].

Traditionally, the supraclavicular block was avoided due to the higher incidence of complications (inadvertent intravascular injection, pneumothorax) that occurred with the use of nerve stimulator and paresthesia technique. However, it has seen an upsurge in recent years as the use of ultrasound guidance has improved patient safety. The use of ultrasound is associated with rapid onset of sensory block, fewer needle attempts, increased block efficacy, and reduced incidence of vascular complications [2].

Ropivacaine is an aminoamide local anesthetic that is less lipophilic than bupivacaine. It causes lower cardiac and central nervous system (CNS) toxicity when compared to other long-acting local anesthetics [3]. Antinociceptive effects of magnesium are attributed to the regulation of calcium influx into the cell and antagonism of the N-methyl-D-aspartate (NMDA) receptors [4]. Magnesium is known to mediate antihypertensive effects when used in anesthesia. Its use through various routes has been studied - intravenous (IV), subarachnoid, epidural, and caudal - to enhance the analgesic potency. While its effect on peripheral nerve blocks has been highlighted, the verdict in the current literature is mixed [5]. In this study, we aimed to compare the efficacy of magnesium sulfate administered IV versus perineurally and as an additive to ropivacaine for supraclavicular brachial plexus block.

## Materials and methods

This prospective randomized study was conducted in the Department of Anesthesiology at Rajarajeswari Medical College and Hospital, Bangalore, Karnataka affiliated to Dr. M.G.R Educational and Research Institute University, Chennai, India, and spanned a period of six months from January 2024 to June 2024. The study aimed to compare the efficacy of magnesium sulfate administered intravenously versus perineurally as an additive for ropivacaine in supraclavicular brachial plexus block for surgeries involving both-bone forearm fractures. Apart from assessing the duration and onset of sensory-motor blockade, total doses of rescue analgesic required, hemodynamic parameters, and other adverse effects, if any, were also analyzed.

After obtaining approval from the Institutions Ethical Committee, Rajarajeswari Medical College and Hospital (vide letter no - IEC/228/2023), this study was registered in the Clinical Trial Registry of India (CTRI/2023/11/060211). A written informed consent was obtained from all the patients. Fifty patients with the American Society of Anesthesiologists (ASA) grades I and II, aged between 20 and 60 years, who underwent surgeries involving both-bone forearm fractures were recruited. Patients with local anesthetic hypersensitivity, bleeding disorders, seizure disorders, neurological diseases, severe hepatorenal diseases, uncontrolled diabetes mellitus, pregnant and lactating women, and patients unwilling to participate were excluded from the study. 

A detailed pre-anesthetic evaluation was done. Patients were explained and educated about the procedure and taught how to interpret the 11-point visual analog scale (VAS) (graded from 0 = no pain to 10 = maximum pain). Fasting guidelines were followed (nil by mouth for six hours). Oral alprazolam 0.25 mg and pantoprazole 40 mg were administered the night before surgery.

Patients were randomly allocated by the sealed opaque envelope method into two groups: Group IV Mg and Group perineural Mg based on a computer-generated code. On arrival of the patients to the operation theatre, an IV line was secured with an 18 G cannula in the unaffected limb, and IV fluids were given according to the requirement. Group IV Mg patients received an injection of ropivacaine 0.75% 20 ml along with 2 mL of normal saline perineurally (total 22 ml) and an injection of magnesium sulfate 150 mg in 100 ml of 0.9% normal saline IV 30 minutes before supraclavicular brachial plexus block. Group Perineural Mg patients received an injection of ropivacaine 0.75% 20 ml with 150 mg of magnesium sulfate (2 ml) perineurally (total 22 ml) and 100 ml of 0.9% normal saline IV for 30 minutes before undergoing supraclavicular brachial plexus block.

Standard ASA monitors were applied, and baseline pulse rate, noninvasive blood pressure, oxygen saturation, and ECG were recorded. The patients were positioned supine with the head turned to the opposite side. An ultrasound device (M-Turbo USG Fujifilm SonoSite, Inc., Bothell, WA) was used. Under aseptic precautions, 2% lignocaine was infiltrated in the supraclavicular fossa after confirming the area of injection which was scanned using a linear probe. The subclavian artery, first rib, pleura, and brachial plexus cluster were identified. Subsequently, a 23-gauge spinal needle was introduced from the lateral to medial direction using an in-plane technique. The needle was advanced towards the area where the lower trunk is commonly situated, which is usually between the first rib inferiorly and the subclavian artery medially. Drug admixture of each group as per the protocol was injected towards all the neural clusters, thereby covering the remaining trunks after negative aspiration of blood.

Block failure was defined as insufficient sensory and unsatisfactory motor blockade after 30 minutes of injecting the studied drugs. The sensory block was considered insufficient if there was no loss of sensation to pinprick, and the motor block was considered as failed if the patients were able to move their fingers. These patients would be administered general anesthesia.

**Figure 1:**
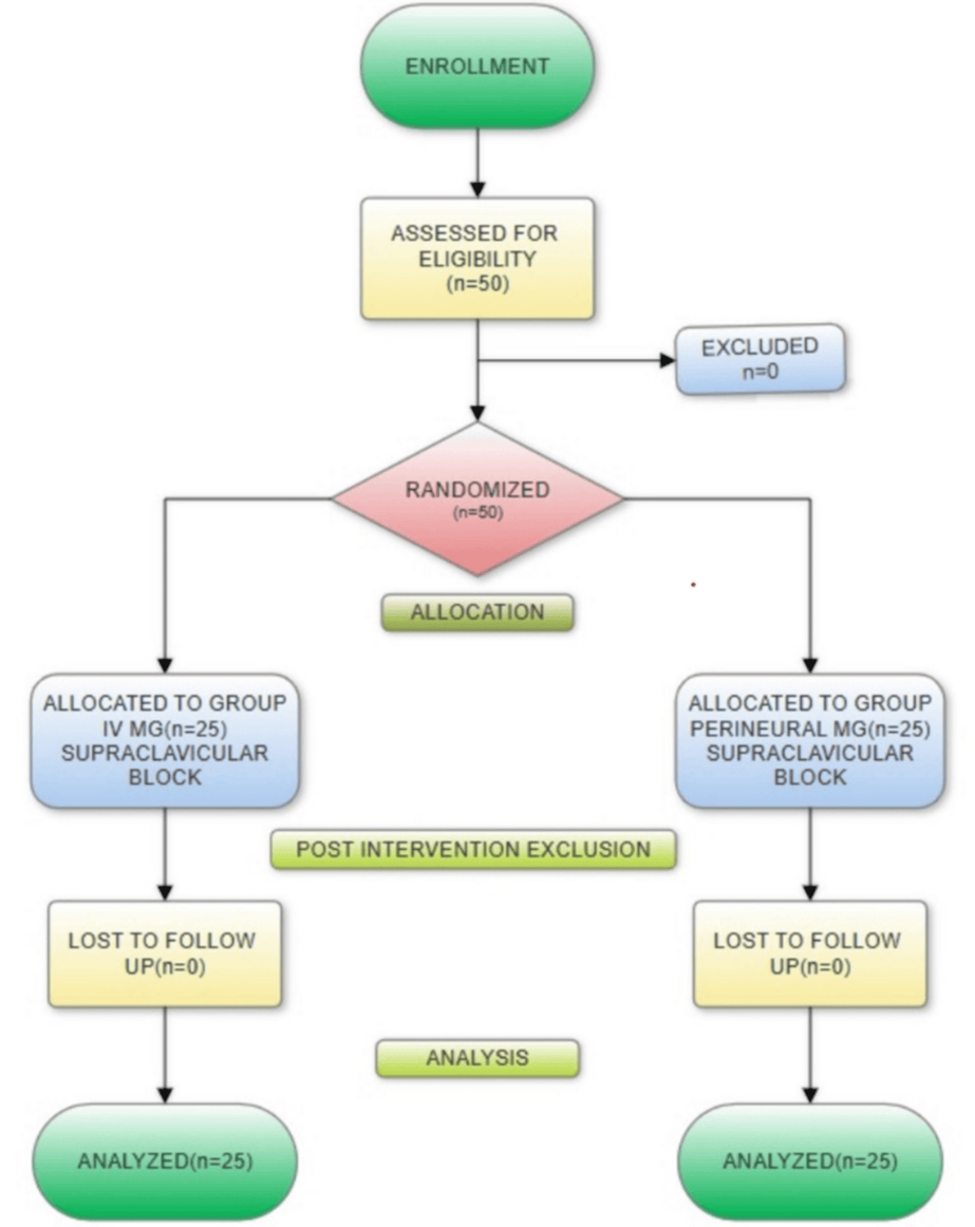
CONSORT flowchart depicting the screening and enrolment of participants CONSORT: Consolidated Standards of Reporting Trials

Both groups were compared regarding the duration of analgesia, which was our primary outcome measure. The onset of both sensory and motor block, the need for rescue analgesics, analgesic and sensorimotor blockade duration, and the total dose of postoperative analgesic required in 24 hours were our secondary outcomes. Patients were asked to note the subjective recovery of sensation and movements, which was then confirmed by an anesthesiologist. Figure 1 presents the CONSORT flowchart illustrating the screening and inclusion of patients.

The following parameters were analyzed

Duration of analgesia was defined as the time between the complete sensory block and the first analgesic request. The onset time for sensory blockade was evaluated as the time interval between the total drug administration and the occurrence of a complete sensory block. The duration of the sensory block was defined as the time interval between the complete sensory block and the total resolution of anesthesia. The onset time for motor block was termed as the time interval between total local anesthetic administration and complete motor blockade. Duration of motor block was computed as the time interval from complete motor block to full recovery of motor function involving hand and forearm.

Sensory block was determined by a pinprick test employing a 3-point scale: 0 = normal sensation, 1 = loss of sensation of pinprick, 2 = loss of sensation of touch [6], whereas the motor blockade was assessed according to the modified Bromage scale on a 3-point score (Grade 0 = normal motor function with full flexion and extension of elbow, wrist, and fingers; Grade 1 = decreased motor strength with ability to move the fingers only; and Grade 2 = complete motor block with inability to move the fingers) [7].

The adverse effects noted were hypotension (i.e., 20% decrease relative to baseline), which was managed by IV ephedrine in dilute concentrations, and bradycardia (HR <50 beats/min), which was countered by IV atropine. Patients who developed nausea, vomiting, and hypoxemia (SpO_2_ <90%) were managed with IV ondansetron and supplemental oxygenation respectively. The need for any additional medications was noted intraoperatively. Hemodynamic parameters were assessed at intervals of 0, 4, 8, 12, and 24 hours postoperatively. Pain was assessed by using VAS. IV paracetamol 1 gm slow IV infusion was used as a rescue analgesic in the postoperative period. The number of doses of IV paracetamol used in 24 hours was noted in both groups and computed appropriately.

Sample size calculation and statistical analysis

Based on a previous study by Sadafule et al. [8], and assuming the difference in the mean duration of analgesia between the perineural magnesium sulfate group and the intravenous magnesium sulfate group as 9.6 hours at a 95% confidence limit and 80% power, and anticipating an approximately 10% possible dropout rate with an alpha of 0.05 and a beta of 0.05, a sample size of 25 was determined for each group. For the primary outcome, the Mann-Whitney U test was used. A p-value <0.05 was considered statistically significant. Data were entered in a Microsoft Excel sheet and were evaluated using SPSS Statistics version 26 software (IBM Corp., Armonk, NY). Categorical data were presented as frequencies and proportions. The unpaired T-test was used as a test of significance for qualitative data. The normality of the continuous data, if compared, was tested by the Kolmogorov-Smirnov test and the Shapiro-Wilk test.

## Results

We recruited 50 patients for the study who were classified into two groups. None of them were excluded from the study. Both groups were comparable in terms of age, weight, height, sex distributions, ASA grading, and duration of surgery (p>0.05) (Table 1).

**Table 1:** Demographic characteristics of the study participants P>0.05: not significant; p<0.05: significant; p<0.001: highly significant. The chi-square test was used for ASA and gender distribution; p<0.05: statistically significant ASA: American Society of Anesthesiologists; SD: standard deviation

Characteristic	Group IV Mg	Group perineural Mg	P-value
Age, years, mean ± SD	45.6 ± 2.7	46.7 ± 3.6	0.67
Weight, kg, mean ± SD	65.9 ± 8.4	67.3 ± 8.92	0.609
Height, cm, mean ± SD	163.8 ± 7.01	165.6 ± 9.48	0.56
Duration of Surgery, min, mean ± SD	95.7. ± 34.27	97.3 ± 32.81	0.82
Variable	Group IV Mg	Group perineural Mg	P-value (chi-square test)
ASA			
Grade 1	27/23	26/24	0.76
Grade 2, n (%)	13 (52%)	12 (48%)	
Gender, n (%)			
Male	10 (40%)	13 (52%)	0.56
Female	15 (60%)	12 (48%)	

Both groups were successfully administered ultrasound-guided supraclavicular brachial plexus block. None of the patients required conversion to general anesthesia in either of the groups. We observed that the duration of analgesia was prolonged in Group perineural Mg (616.48 ± 92.396 minutes) compared to Group IV Mg (459.81 ± 82 minutes) (p<0.001) (Table 2).

**Table 2:** Comparison of the duration of analgesia, onset, duration of block, total analgesic requirement, and VAS score between the groups P-values are obtained using the Mann-Whitney U test. P-value is statistically significant if <0.05; not significant if >0.05 ASA: American Society of Anesthesiologists; SD: standard deviation; VAS: visual analog scale

Variable	Group IV Mg, mean ± SD	Group perineural Mg, mean ± SD	P-value
Duration of analgesia (from zero time), min,	459.81 ± 82.984	616.48 ± 92.396	0.001
Onset of sensory block, min	12.81 ± 3.37	11.29 ± 3.27	0.145
Onset of motor block, min	14.43 ± 3.82	13.29 ± 2.29	0.273
Duration of sensory block, min	426.19 ± 60.87	565.24 ± 91.41	<0.001
Duration of motor block, min,	319.52 ± 58.52	463.81 ± 89.19	<0.001
Total analgesic requirement (paracetamol), gms	1.71 ± 0.463	1.14 ± 0.359	<0.001
VAS score (post-op)			
0	2.1 ± 0.86	2.2 ± 0.62	0.32
4	1.24 ± 0.70	0.71 ± 0.78	0.029
8	2.76 ± 1.09	2.76 ± 1.09	0.027
12	3.57 ± 1.16	3.29 ± 1.65	0.576
24	2.43 ± 1.03	2.38 ± 1.40	0.657

The onset time of both sensory and motor block was comparable between group IV Mg and group Perineural Mg [(11.29 ± 3.27 vs. 12.81 ± 3.37 min) (p<0.145) and (13.29 ± 2.76 min and 14.43 ± 3.82) (p=0.273) respectively]. The mean sensory blockade was prolonged in group perineural Mg when compared to group IV Mg (565.24 ± 91.47 min vs. 426.19 ± 60.87 min). The mean motor block duration was also enhanced in the perineural group (463.81 ± 89.19 min vs. 319.52 ± 58.52 min) (p<0.001) (Table 2). The total postoperative analgesic requirement in group perineural Mg was lower when compared to group IV Mg (p<0.001) (Table 2). The VAS score was significant at the fourth and eighth hour postoperatively (p<0.05) (Table 2).

**Figure 2:**
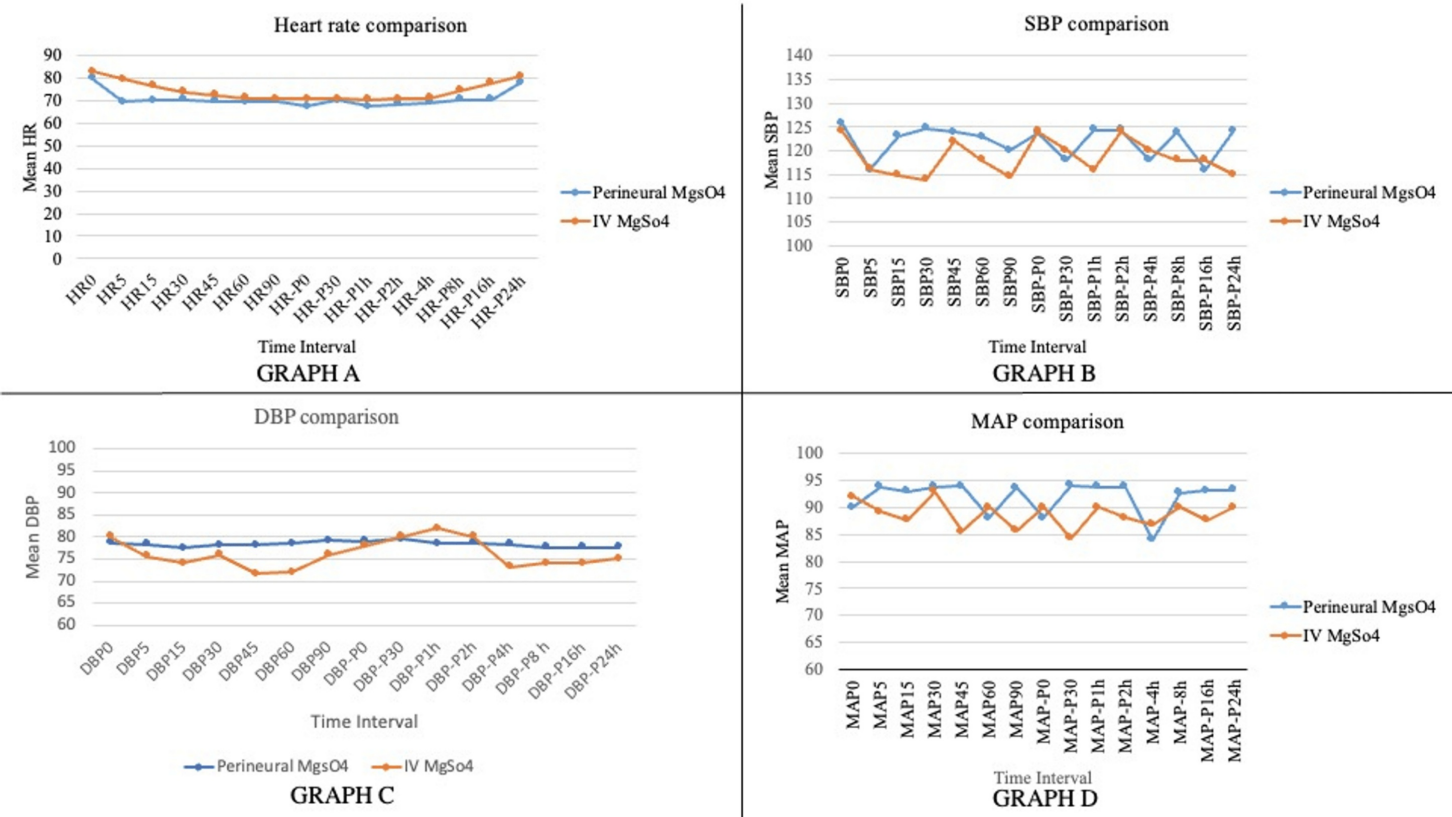
Panel of graphs showing hemodynamic monitoring Graph A: Line diagram showing heart rate comparison between two groups at different periods of follow-up. Graph B: Line diagram showing systolic blood pressure comparison between two groups at different periods of follow-up. Graph C: Line diagram showing diastolic blood pressure comparison between two groups at different periods of follow-up. Graph D: Line diagram showing MAP comparison between two groups at different periods of follow-up DBP: diastolic blood pressure; MAP: mean arterial pressure; SBP: systolic blood pressure

There was no apparent local anesthetic toxicity in either of the groups. The hemodynamic parameters were monitored at intervals of 0, 1, 2, 4, 8, 16, and 24 hours and we did not encounter any significant hemodynamic instability among patients of both groups (Figure 2).

None of the patients experienced any adverse effects such as nausea, vomiting, hypotension, and bradycardia during intraoperative and postoperative periods.

## Discussion

General anesthesia was earlier commonly used for upper limb surgeries. Stress responses associated with laryngoscopy, intubation, nausea, vomiting, and postoperative respiratory depression were the adverse effects observed. Brachial plexus block provides better analgesia in the perioperative period as the upper limb is solely innervated in the procedure [7]. With the advent of ultrasound, which enables the clear visualization of nerve clusters, the supraclavicular brachial plexus block provides excellent analgesia with a lower volume of local anesthetics. Also, it helps avoid accidental intraarticular injection and injury to the pleura. A lower volume of the local anesthetic is required. Ropivacaine was chosen for our study because it has negligible neurological and cardiac toxicity [9].

Magnesium sulfate has the property to enhance the analgesic effect of local anesthetic drugs when used as an adjuvant in regional anesthesia [9]. There are few studies in the literature studies comparing the different routes of magnesium sulfate administration (perineural vs. intravenous) as an adjuvant to different local anesthetics. This prompted us to compare the effects of intravenous and perineural magnesium sulfate in upper limb orthopedics surgeries involving both-bone forearm fractures under ultrasound-guided brachial plexus block. In our study, the efficacy between perineural and intravenous magnesium sulfate in supraclavicular brachial plexus block was compared. The duration of analgesia was prolonged in the perineural group. A minimal dose of rescue analgesic was consumed during the postoperative period in the perineural group.

The sensory and motor blockade commencement in both groups was comparable. The duration of sensory and motor blockade was also prolonged in patients who received perineural magnesium sulfate. We did not come across any hemodynamic instability or adverse effects in either of the groups. There are limited studies in the literature comparing perineural vs. intravenous low-dose magnesium sulfate as an adjuvant to ropivacaine in upper-limb orthopedic surgeries. Our decision to choose 150 mg of magnesium sulfate in this study was based on the concept that magnesium mediates a lot of physiological processes at higher doses and the patients may develop adverse effects as we did not measure preoperative serum magnesium levels in any of our patients.

Sadafule et al. [8] have conducted a study similar to ours, where they compared perineural magnesium sulfate and intravenous magnesium sulfate as an adjuvant to bupivacaine in the supraclavicular block under ultrasound guidance for orthopedic surgeries involving the upper limb. They found that the duration of analgesia was significantly higher in the group that received perineural magnesium sulfate when compared to the intravenous group (p<0.05). Our findings partially correlated with their observations. On the contrary, they observed that the onset of sensory blockade in perineural block was delayed. This may be attributed to dose alterations, as they used a higher dose of 50 mg/kg in 100 ml of normal saline through the intravenous route and 250 mg perineurally. We found that though the onset of sensory and motor blockade was comparable between both groups, the duration of sensory-motor blockade was prolonged in the perineural group.

Since we did not measure the serum magnesium levels preoperatively, a low dose (150 mg) of magnesium sulfate was used to avoid any adverse effects. We used the same concentration in both groups, intending to maintain equipotency as there are no equipotent dosage protocols between perineural and intravenous magnesium to date in the literature. There are various conflicting reports regarding the effectiveness of intravenous vs. perineural magnesium as an adjuvant to ropivacaine in upper limb surgeries. This was postulated by Khanal et al. [10], as their observations revealed that both perineural and intravenous magnesium sulfate as adjuvants to ropivacaine are equally efficacious in prolonging the duration of analgesia. They had used 50 mg/kg of magnesium sulfate through the intravenous route. Thus, sensory and motor duration was significantly prolonged in the perineural group as compared to the intravenous group. In our study, the reason for the difference in analgesic efficacy between the groups is still ambiguous.

A study by Dogru et al. [11] observed a quicker onset of the sensory and motor blockade in patients who received magnesium sulfate as an adjuvant to levobupivacaine in axillary brachial plexus block involving arteriovenous fistula surgeries in chronic kidney disease patients(p<0.05). Olapour et al. [12] opined that magnesium sulfate considerably lengthened the pain-free period when used as an adjuvant to lignocaine in the supraclavicular block. This study also emphasized the importance of perineural magnesium sulfate as an adjuvant to a local anesthetic.

There are studies in the literature comparing intrathecal and intravenous magnesium sulfate. Arvind et al. [13] and Samir et al. [14] compared intravenous and intrathecal magnesium sulfate for postoperative analgesia and observed that the analgesic duration was significantly prolonged in the group that received intravenous magnesium sulfate when compared to control and intrathecal groups. Magnesium sulfate has also been used as an adjuvant to local anesthetics in other fascial plane blocks. Olapour et al. [12]. observed a noticeably longer duration of analgesia after using 150 mg of magnesium sulfate as an adjuvant in TAP block during laparoscopic cholecystectomy, which also prompted us to choose magnesium sulfate of the same dosage in our study.

Our findings revealed that patients who received magnesium sulfate perineurally not only had a longer duration of analgesia but also had prolonged sensory and motor blockade and required fewer rescue analgesic doses in the first 24 hours postoperatively than patients who received it intravenously.

Limitations

Our study has a few limitations. Primarily, only ASA 1 and 2 patients were recruited in this study. We did not consider the placement of a catheter, which would serve as a tool for providing analgesia for a duration beyond 24 hours. We did not measure serum magnesium levels for any of the patients in either of the groups. We did not include a control group as it was not feasible, and we recommend that future studies include a control group. We did not assess satisfaction and sedation scores for any of the patients. Further research involving a huge cohort is warranted to substantiate our findings. Though we used similar, low doses of magnesium sulfate (150 mg) in both perineural and intravenous routes, further studies need to be carried out to validate our observations by using different dosage protocols of magnesium sulfate. This would help determine the optimal and safest dose of magnesium sulfate in clinical practice.

## Conclusions

Our findings showed that magnesium sulfate is associated with a significant duration of analgesic effect when administered perineurally along with ropivacaine in supraclavicular brachial plexus block when compared to intravenous route of administration. It also enhances the quality of the block, and hence we propose that it is a potential adjuvant to local anesthetics in brachial plexus block. We recommend the widespread use of magnesium sulfate as an adjuvant to local anesthetics in ultrasound-guided brachial plexus block.

## References

[1] Halsted WS (1885). Practical comments on the use and abuse of cocaine: suggested by its invariably successful employment in more than a thousand minor surgical operations. NY Med J.

[2] Bruce BG, Green A, Blaine TA, Wesner LV (2012). Brachial plexus blocks for upper extremity orthopaedic surgery. J Am Acad Orthop Surg.

[3] Vainionpää VA, Haavisto ET, Huha TM (1995). A clinical and pharmacokinetic comparison of ropivacaine and bupivacaine in axillary plexus block. Anesth Analg.

[4] Sirvinskas E, Laurinaitis R (2002). Use of magnesium sulfate in anesthesiology (Article in Lithuanian). Medicina (Kaunas).

[5] Agrawal A, Agrawal S, Payal YS (2014). Effect of continuous magnesium sulfate infusion on spinal block characteristics: a prospective study. Saudi J Anaesth.

[6] Nallam SR, Chiruvella S, Karanam S (2017). Supraclavicular brachial plexus block: Comparison of varying doses of dexmedetomidine combined with levobupivacaine: a double-blind randomised trial. Indian J Anaesth.

[7] Dhanger S, Vaidyanathan B, Rajesh IJ, Vinayagam S, Bahurupi Y, Vimalraj D (2016). Efficacy of low dose intravenous dexamethasone for prolongation of analgesia in supraclavicular block: randomized controlled trial. Indian J Pain.

[8] Sadafule NN, Deshpande J, Patil K (2022). Comparing the effects of perineural magnesium sulphate with intravenous magnesium sulphate as an adjuvant to bupivacaine in USG-guided supraclavicular block. Arch Anesth Crit Care.

[9] Do SH (2013). Magnesium: a versatile drug for anesthesiologists. Korean J Anesthesiol.

[10] Khanal D, Sah BP, Bhattarai B (2023). The effectiveness of magnesium sulphate intravenous bolus or added as an adjunct to ropivacaine for brachial plexus block in upper limb orthopaedic surgeries. Int J Innov Sci Res Technol.

[11] Dogru K, Yildirim D, Ulgey A, Aksu R, Bicer C, Boyaci A (2012). Adding magnesium to levobupivacaine for axillary brachial plexus block in arteriovenous fistule surgery. Bratisl Lek Listy.

[12] Olapour AR, Mohtadi AR, Soltanzadeh M, Ghomeishi A, Akhondzadeh R, Jafari M (2017). The effect of intravenous magnesium sulfate versus intravenous sufentanil on the duration of analgesia and postoperative pain in patients with tibia fracture. Anesth Pain Med.

[13] Kumar A, Chaudhary UK, Kansal D, Rana S, Sharma V, Kumar P (2016). Comparison of intravenous magnesium sulphate with intrathecal magnesium sulphate for postoperative analgesia in orthopaedic patients undergoing extracapsular hip fracture surgery. Int J Basic Clin Pharmacol.

[14] Samir EM, Badawy SS, Hassan AR (2013). Intrathecal vs intravenous magnesium as an adjuvant to bupivacaine spinal anesthesia for total hip arthroplasty. Egypt J Anaesth.

